# Mechanical Lymphatic Drainage (RAGodoy®): Literature Review

**DOI:** 10.7759/cureus.21263

**Published:** 2022-01-15

**Authors:** Jose Maria Pereira de Godoy, Maria de Fatima Guerreiro Godoy, Henrique Jose Pereira de Godoy

**Affiliations:** 1 Cardiology and Cardiovascular Surgery, São José do Rio Preto School of Medicine (FAMERP), São José do Rio Preto, BRA; 2 Angiology and Vascular Surgery, Clínica Godoy, São José do Rio Preto, BRA; 3 Occupational Therapy, São José do Rio Preto School of Medicine (FAMERP) and Clínica Godoy, São José do Rio Preto, BRA; 4 General Surgery, São José do Rio Preto School of Medicine (FAMERP) and Clínica Godoy, São José do Rio Preto, BRA; 5 General Practice, Clínica Godoy, São José do Rio Preto, BRA

**Keywords:** lymphatic drainage, mechanical, treatment, edema, lymphedema

## Abstract

Lymphatic drainage is the main form of therapy for lymphedema, as it affects the pathophysiology of this clinical condition. The two main objectives of lymphatic drainage are the formation and drainage of lymph. In recent years, Godoy & Godoy developed a novel concept of mechanical lymphatic drainage involving a device denominated RAGodoy®, which performs passive exercises of the lower and upper limbs as a form of lymphatic drainage. The aim of the present study was to address the concept of this therapy as well as perform a literature review on its forms of use and the results obtained. All studies analyzed show that this technique used as monotherapy enables the treatment of lymphedema, but superior results are achieved when combined with compression mechanisms.

## Introduction and background

Lymphedema is a clinical condition that leads to a specific type of edema, the cause of which is a failure in the formation or drainage of lymph [1-2]. Clinical staging takes into account the manifestation of the edema and the deformities observed. In grade I lymphedema, the swelling appears during the day and in grade II, the patient awakens with edema in the morning, which normally worsens during the day. Grade III lymphedema is similar to grade II but more advanced and with worse deformities [2]. Severity may be mild with a volume increase of up to 20% (compared to the normal contralateral leg), medium with increases of between 20% and 40%, or severe with increases of more than 40% [3]. An association of therapies is recommended to treat lymphedema with lymph drainage, compression mechanisms, and exercising [4]. One of the advances in the treatment of lymphedema is mechanical lymphatic drainage using a device denominated RAGodoy®, which performs passive plantar flexion and extension or flexion-extension of the arm [5]. This device has been adapted and improved over the years, giving it new forms of use. The aim of the present study was to address the concept of this therapy as well as perform a literature review on its forms of use and the results obtained.

## Review

RAGodoy® performance

Physiological lymphatic drainage performed by the body itself uses mechanisms that promote a pressure differential in the intravascular and extravasated fluid environment. The main pressures used by the lymphatic vessels are the pressures vis front, vis latere, and the contraction of the lymphangions. The vis latere pressure is generated by the compression of the artery under the lymphatic during cardiac systole, muscle activity, and peristalsis, and the vis-front pressure is caused on the forehead during breathing movements [6].

The space between one valve and another in the lymphatic vessels constitutes a functional unit in which it performs a “lymph pumping” effect similar to the heart. It follows the same principles of cardiac contraction where filling triggers contraction as well as external compression.

 Another way to stimulate the venous and lymphatic displacement is through muscular work that performs an external compression having an effect of “pressure vis latere.” Muscle work is essential to help the venous and lymphatic systems overcome the “negative” effect of gravitational pressure.

Gravitational pressure can have an opposite or favorable effect on lymphatic drainage, and this depends on the individual's posture. Lifting the limb favors this return, and the bipedal position makes it difficult to return.

**Figure 1:**
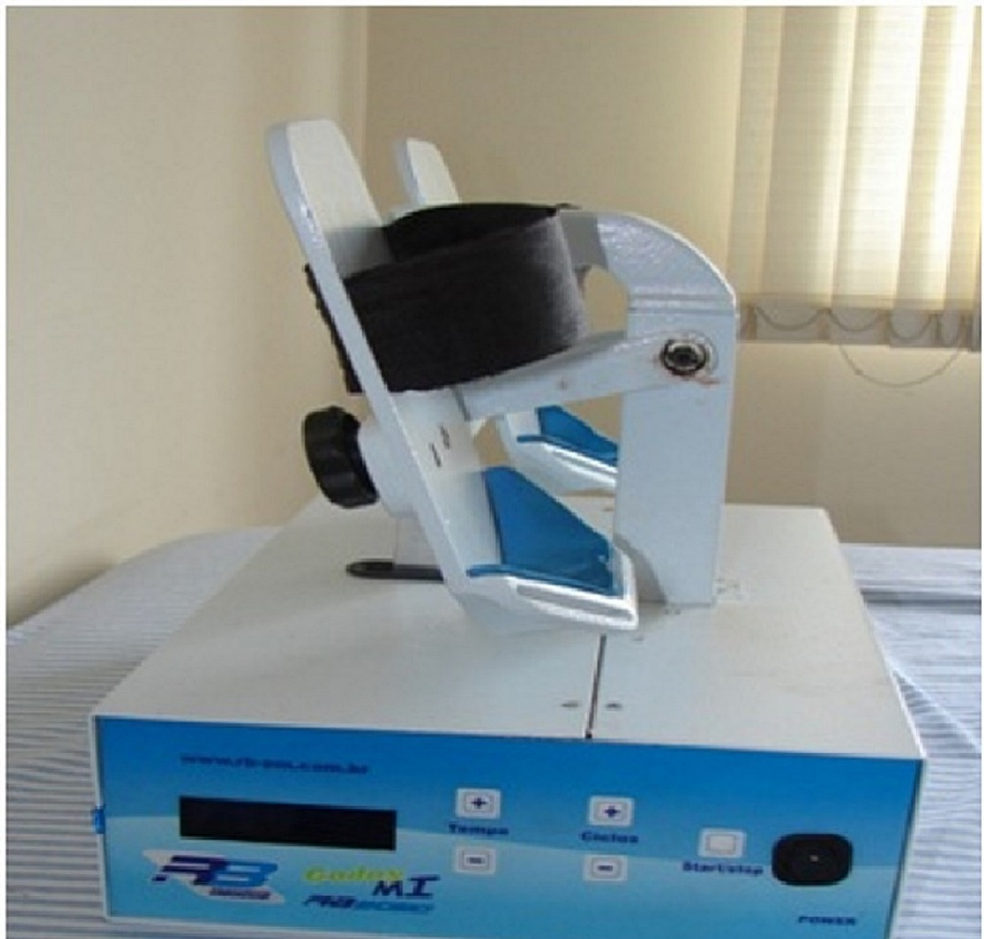
The RAGodoy® apparatus for legs – side view

**Figure 2:**
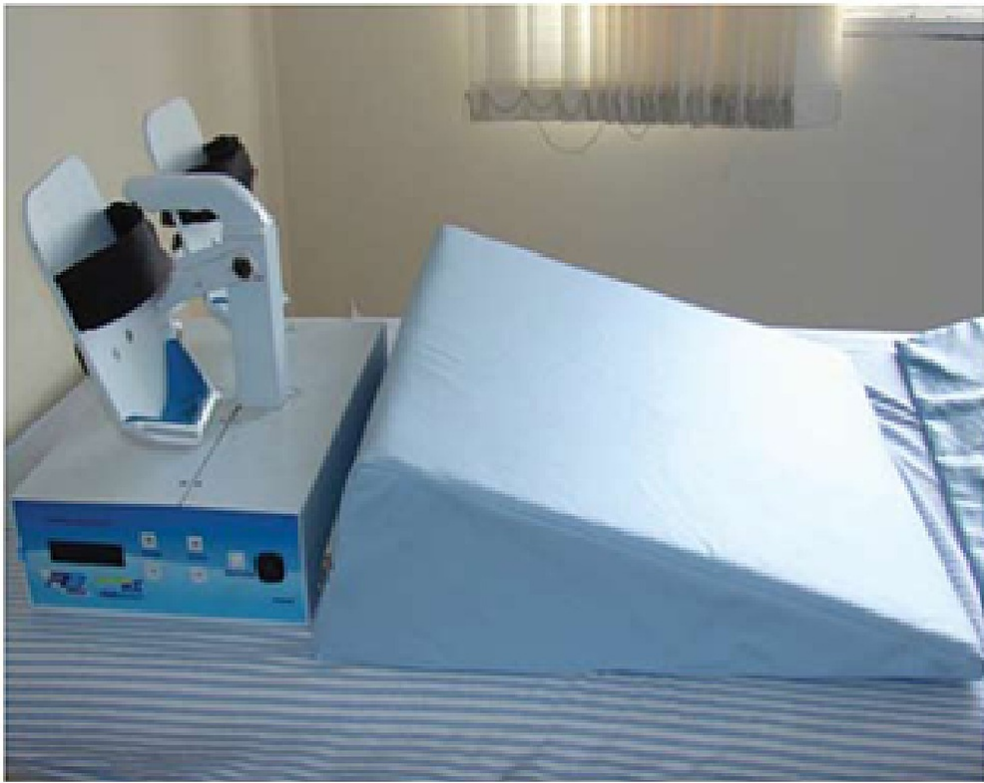
The RAGodoy® apparatus for lower limbs with a foam support wedge to help to bear the weight of the lower limb and to reduce the effect of gravity

**Figure 3:**
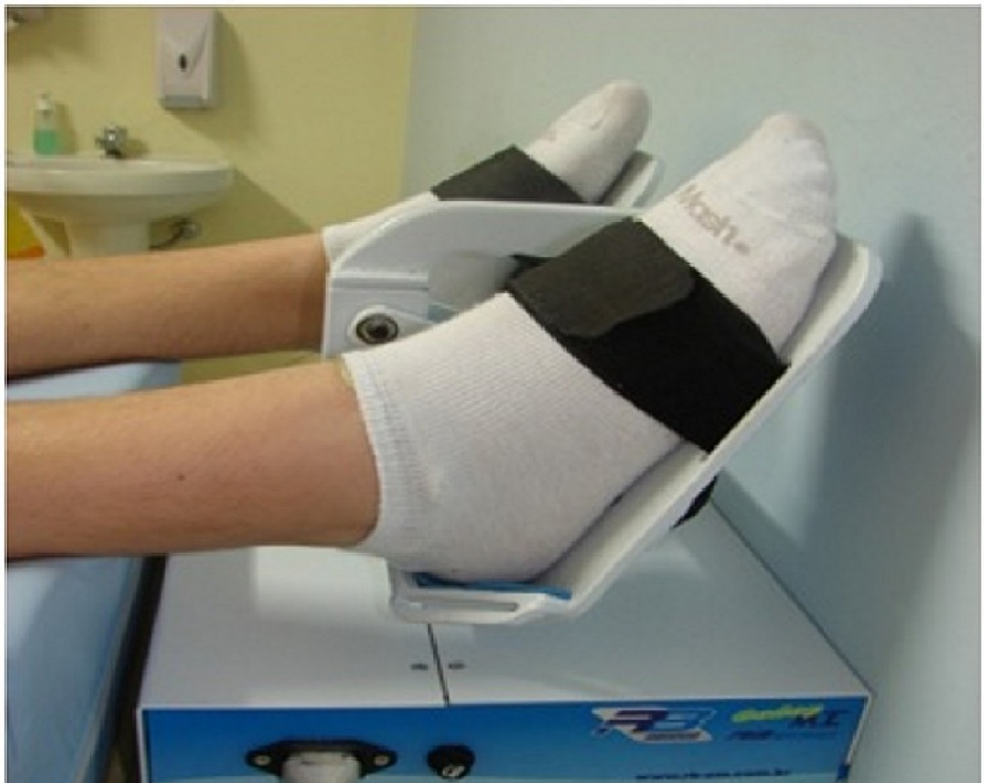
The RAGodoy® apparatus for lower limbs with the feet in position, ready to start treatment

**Figure 4:**
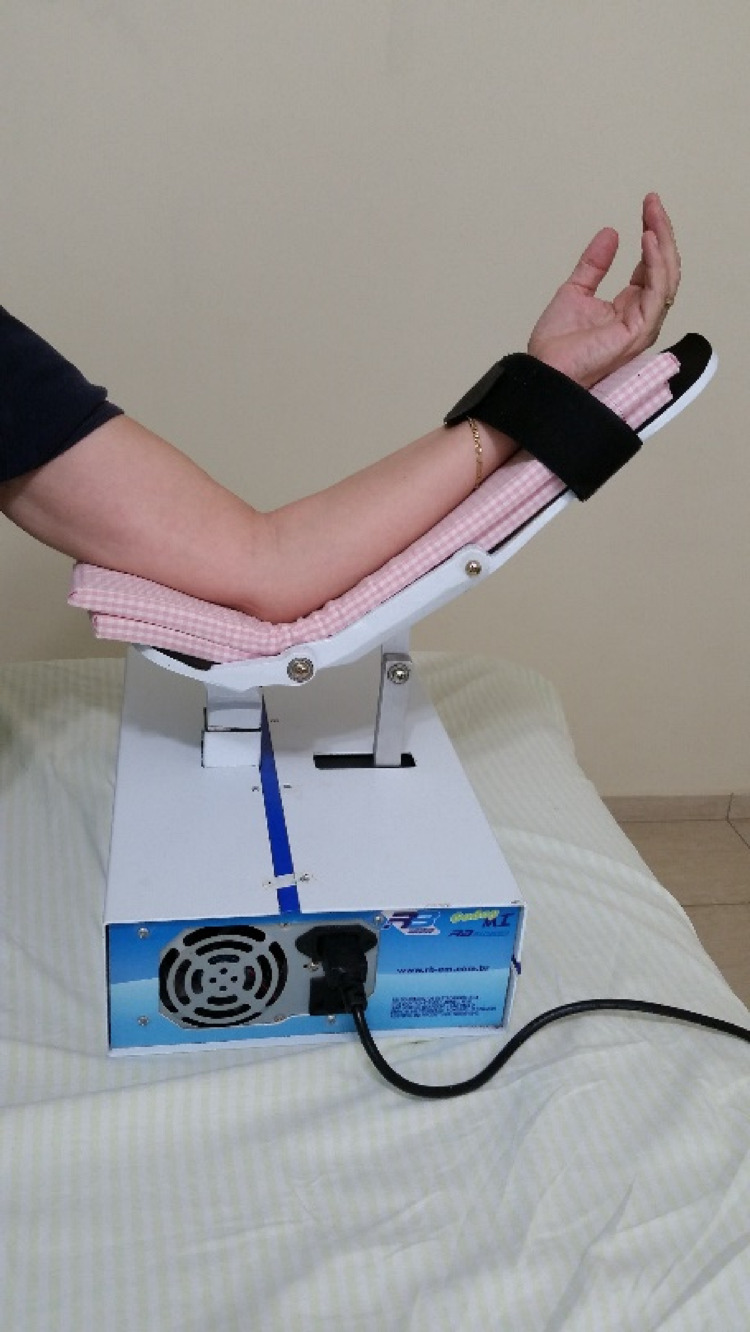
RAGodoy® for upper limbs

Knowledge of these physiological drainage mechanisms allowed the development of an electromechanical device known in Brazil as RAGodoy®, which performs plantar flexion and extension (Figures 1-3) and extension movements of the elbow (Figure 4). Thus, there is a “passive” stimulus of the limb's muscle work, facilitating the venolymphatic return, which constitutes a passive exercise that was adapted to the treatment of lymphedema. The performance of the RAGodoy® device of the lymphedema treatment has been used and evaluated over the years. The RAGodoy® device represents a new concept in lymphatic drainage, as it reproduces physiological movements that facilitate and encourage drainage systems.

First, the device developed was for lower limbs with the aim of reproducing the physiological movements of lymphovenous drainage by muscle contraction, thereby simulating the movements of walking, including plantar flexion and extension, but in the supine position. Thus this appliance, with the help of gravity, physiologically but passively, increases lymph drainage. In fact, this is a passive exercise. The second time, the apparatus was developed for improving lymphatic drainage in the upper limbs; this is the same mechanism reproducing the physiological movements of lymphovenous drainage by muscle. The RAGodoy® device for the upper limbs works by contraction of the biceps and triceps muscles and passive movements, with 15 cycles (movements-flexion-extension-elbow) per minute.

Scientific study

The scientific literature has 40 studies addressing the use of this device (RAGodoy®) as monotherapy (10 studies) or combined with other forms of treatment for lymphedema of the lower and upper limbs. The first study was published in 2004 and involved an evaluation with lymphoscintigraphy, which revealed that the device stimulates the mobilization of macromolecules [5]. The second study was published in 2005 and addressed a combination of therapies [7]. The third study, published in 2006, evaluated the reduction in the volume of edema [8]. A fourth study evaluated changes in the venous pressure of the hallux during the action of the device, demonstrating that it stimulates venous return [9]. In 2009, one study showed a significant reduction in the volume of the edema during one hour of treatment and another study showed a reduction in skin fibrosis [10-11].

In 2011, the RAGodoy® device was investigated in a combination of therapies for the treatment of cellulite [12]. In 2012, the device was used as intensive treatment (eight hours per day) combined with a compression mechanism, revealing the possibility of large reductions in volume (approximately 50% of the edema) in a period of five days [13]. In 2013, another assessment of the reduction in volume was published involving analyses of volumetry and bioimpedance, which confirmed the previous findings [14-15]. The device was employed for the treatment of lipedema associated with cellulite, demonstrating beneficial effects [16].

In 2014, the RAGodoy® device was used on a child with lymphedema and intellectual disability, and another study showed a significant reduction in edema when combined with a compression mechanism [17-18]. In 2016, the use of the device was evaluated regarding the clinical evolution of the skin, the intensive treatment of lymphedema associated with Klippel-Trenaunay syndrome, and in the preoperative period of orthopedic surgery to achieve a reduction in lymphedema [19-21].

In 2017, studies were conducted evaluating the use of the RAGodoy® device in the treatment of cellulite, the treatment of lymphedema as a sequela of poliomyelitis, the intensive treatment of elephantiasis in a child, and the maintenance of the results for treatment for lymphedema [22-25]. Moreover, its results in the intensive treatment of lymphedema were assessed in the same year, considering the phase angle of bioimpedance [26].

In 2018, the RAGodoy® device was evaluated regarding the change in body mass index during intensive treatment, the treatment of post-traumatic lymphedema, and the mobilization of fluids during intensive treatment [27-29]. In 2019, studies were conducted assessing the use of the device in the intensive treatment of lymphedema in occupational rehabilitation, the assessment of its results using impedance and reactance, its effects on joint mobility, and its effects on the maintenance of the results of the treatment of lymphedema [30-33]. In 2020, a study was conducted evaluating the effects of the device on the reduction in edema in 409 patients submitted to intensive treatment with a combination of therapies [34]. In 2021, a new study was conducted assessing the effects of the combination of therapies on the mobilization of body fluids [35].

The upper limb device has had slower evolution. The first study was published in 2009 and showed a reduction in volume when the device was used as monotherapy [36]. In 2011, bioimpedance was used to assess the results of the device as monotherapy, revealing a reduction in edema [37]. In 2012, two new studies reproduced these evaluations and confirmed the effectiveness of the device [38-39].

In 2013, a study was conducted evaluating three hours of treatment, revealing a greater reduction in the first hour and a smaller reduction in the third hour [40]. This finding suggested the need for changes to adapt to longer periods of intensive treatment, as performed with the device used on the lower limbs, and new evaluations were performed [41]. In 2021, a study was conducted evaluating the device for intensive treatment combined with compression mechanisms involving the use of a laced sleeve made of grosgrain fabric and bandages, cervical lymphatic therapy, and manual lymphatic therapy using the Godoy & Godoy method, which enabled a reduction in the volume of the limb by approximately 50% in five days (40 hours) of intensive treatment [42].

These publications show the reproducibility of treatment and the possibility of using the RAGodoy® device as monotherapy or in combination with other therapies. The device constitutes an important mechanical lymphatic drainage mechanism. Indeed, intensive treatment (eight hours per day) is nearly impossible without the use of the device, making it an important contribution to the treatment of lymphedema. The use of the device as monotherapy is possible for milder forms of lymphedema, but the results are superior when used in combination with compression mechanisms.

## Conclusions

All studies analyzed show that the use of the RAGodoy® device for mechanical lymphatic drainage for lymphedema treatment of the lower limbs and upper limbs considerably assists in both the treatment and the maintenance of the results. Another point in relation to these devices is that mechanical lymphatic drainage as monotherapy reduces edema, but better results are achieved when combined with compression mechanisms.

## References

[1] Lee BB, Antignani PL, Baroncelli TA (2015). IUA-ISVI consensus for diagnosis guideline of chronic lymphedema of the limbs. Int Angiol.

[2] Executive Committee of the International Society of Lymphology (2020). The diagnosis and treatment of peripheral lymphedema: 2020 consensus document of the International Society of Lymphology. Lymphology.

[3] Maclellan RA, Zurakowski D, Voss S, Greene AK (2017). Correlation between lymphedema disease severity and lymphoscintigraphic findings: a clinical-radiologic study. J Am Coll Surg.

[4] Barufi S, Pereira de Godoy HJ, Pereira de Godoy JM, Guerreiro Godoy MF (2021). Exercising and compression mechanism in the treatment of lymphedema. Cureus.

[5] de Godoy JM, Godoy Mde F (2004). Development and evaluation of a new apparatus for lymph drainage: preliminary results. Lymphology.

[6] Guyton AC, Hall JE The microcirculation and lymphatic system: capillary fluid exchange, interstitial fluid, and lymph flow. Guyton and Hall Textbook of Medical Physiology.

[7] Godoy JMP, Godoy MFG (2005). New apparatus for mechanical lymphatic drainage in association of therapies in the treatment of lymphedema. Acta Phlebol.

[8] JMP Godoy, MFG Godoy (2006). Development and evaluation of an apparatus for oedema drainage. Angiología.

[9] Godoy JMP, Godoy MFG, Batigália F, Xavier MIG (2008). Dynamic evaluation of the venous pressure during passive plantar flexion and dorsiflexion exercises with the RAGodoy® apparatus. IJPMR.

[10] Siqueira Da Silva K, Karan MG (2009). Volumetric alterations utilizing the RAGodoy® device to treat lymphedema of the lower extremities. J Phlebol Lymphology.

[11] Pereira de Godoy JM, Braile DM, de Fátima Guerreiro Godoy M (2008). Lymph drainage in patients with joint immobility due to chronic ulcerated lesions. Phlebology.

[12] de Godoy JM, de Godoy Mde F (2011). Treatment of cellulite based on the hypothesis of a novel physiopathology. Clin Cosmet Investig Dermatol.

[13] Pereira de Godoy JM, Gonçalves IP, Barufi S, Godoy Mde F (2012). Large reduction in volume with the intensive treatment of lymphedema: reduction of fluids?. Int J Angiol.

[14] Brigídio PAF, Buzato E, Barufi S, Guimarães TD, Pinto RL, Libanore D (2013). Volumetric evaluation after treatment using the RAGodoy® apparatus in patients with lower limb lymphedema. Arq Ciênc Saúde.

[15] Brigidio PAF, Godoy JMP, Pinto RL, Guimarães TD, Godoy MFG (2013). Reducing the volume of lower limb lymphedema with mechanical lymphatic drainage RAGodoy® evaluated by bioimpedance. Angiol Cir Vasc.

[16] de Godoy JM, Barufi S, Godoy Mde F (2013). Lipedema: is aesthetic cellulite an aggravating factor for limb perimeter?. J Cutan Aesthet Surg.

[17] de Godoy JM, Sanchez AP, Zucchi Libanore D, Guerreiro Godoy Mde F (2014). Adaptations in the treatment of congenital lymphedema centered on the quality of life. Case Rep Med.

[18] de Godoy JM, Lopes Pinto R, Pereira de Godoy AC, de Fátima Guerreiro Godoy M (2014). Synergistic effect of adjustments of elastic stockings to maintain reduction in leg volume after mechanical lymph drainage. Int J Vasc Med.

[19] Pereira de Godoy HJ, Budtinger Filho R, Godoy MF, de Godoy JM (2016). Evolution of skin during rehabilitation for elephantiasis using intensive treatment. Case Rep Dermatol Med.

[20] de Godoy JM, Río A, Domingo Garcia P, de Fatima Guerreiro Godoy M (2016). Lymphedema in Klippel-Trenaunay syndrome: is it possible to normalize?. Case Rep Vasc Med.

[21] Guerreiro Godoy MF, Pereira de Godoy LM, Lopes Pinto R, Pereira de Godoy JM (2016). Preoperative preparation of a patient with grade II leg Lymphedema for his third hip replacement surgery. Int J Surg Case Rep.

[22] de Godoy JM, de Godoy AC, Godoy MF (2017). Considering the hypothesis of the pathophysiology of cellulite in its treatment. Dermatol Reports.

[23] Guerreiro Godoy M, Pinto RL, Godoy JMP (2017). Intensive treatment of lymphedema in a patient with a sequela of polio. Int J Physiother.

[24] Godoy ACP, Troitino RO, Godoy MFG, Godoy JMP (2017). Intensive treatment of elephantiasis in a child: a case report. Medical Case Report.

[25] Pereira de Godoy JM, Pereira de Godoy HJ, Lopes Pinto R, Facio FN Jr, Guerreiro Godoy MF (2017). Maintenance of the results of stage ii lower limb lymphedema treatment after normalization of leg size. Int J Vasc Med.

[26] Godoy JMP, Fortuny EI, Godoy HJP, Godoy MFG (2017). Phase angle in the assessment of intensive outpatient treatment of primary lower limb lymphedema. J Phlebol Lymphology.

[27] Godoy HJP, Ataç D, Godoy MFG (2018). Changes in body mass and fat mass indexes in patients submitted to intensive treatment for lymphedema. Ann Med Health Sci Res.

[28] Pereira de Godoy AC, Ocampos Troitino R, de Fátima Guerreiro Godoy M, Pereira de Godoy JM (2018). Lymph drainage of posttraumatic edema of lower limbs. Case Rep Orthop.

[29] de Godoy JM, Pereira de Godoy HJ, Gracino de Marqui T, Spessoto LC, Godoy MF (2018). Mobilization of fluids in the intensive treatment of primary and secondary lymphedemas. ScientificWorldJournal.

[30] de Godoy JM, de Godoy HJ, de Godoy AC, Godoy MF (2019). Elephantiasis and directed occupational rehabilitation. Case Rep Vasc Med.

[31] Godoy JMP, Godoy HJP, Godoy MFG (2019). Evaluation of impedance and reactance in the intensive treatment of lymphoedema. J Clin Diagn Res.

[32] Godoy JMP, Godoy LMP, Godoy ACP, Pinto RL, Godoy MFG (2019). Gain in joint mobility during intensive treatment of lymphedema using Godoy method. Acta Phlebol.

[33] Godoy JMP, Godoy HJP, Godoy ACP, Godoy MFG (2019). Elephantiasis and maintenance of results following intensive treatment. J Clin Diagn Res.

[34] Pereira de Godoy JM, Guerreiro Godoy MF, Barufi S, Pereira de Godoy HJ (2020). Intensive treatment of lower-limb lymphedema and variations in volume before and after: a follow-up. Cureus.

[35] Godoy MFG, Godoy JMP, Godoy LMP, Godoy HJP, Ramos RR (2021). Intensive treatment of lymphedema and mobilization of liquids in body segments. Med Sci.

[36] Bordin NA, Godoy MFG, Godoy JMP (2009). Mechanical lymphatic drainage in the treatment of arm lymphedema. Indian J Cancer.

[37] Libanore D, Buzato E, Barufi S, Guimarães TD, Carvalho ECM, Brigido PAF (2011). Bioimpedance assessment of edema in patients with mastectomy-related lymphedema treated by mechanical lymph drainage using the RAGodoy® device. J Phlebol Lymphology.

[38] Godoy MFG, Guimarães TDG, Barufi S, Lopes R, Brigidio PAF (2012). Drenaje linfático mecánico en el tratamiento de pacientes pos-mastectomía - estudio piloto [Article in Spanish]. Flebología Y Linfología - Lecturas Vasculares.

[39] de Godoy JM, Godoy Mde F (2013). Evaluation of a new approach to the treatment of lymphedema resulting from breast cancer therapy. Eur J Intern Med.

[40] Bordin NA, Godoy JMP, Barufi S, Godoy MFG (2013). Pilot study to evaluate an apparatus for mechanical lymph drainage. Acta Angiol.

[41] Godoy MFG, Libanori D, Pinto RL, Godoy JMP (2013). Intensive treatment of breast cancer-related lymphedema in patients with neurological injuries [Article in Portuguese]. Acta Fisiatr.

[42] Godoy ACP, Godoy MFG, Godoy LMP, Godoy HJP, Godoy JMP (2021). Intensive treatment for upper limb lymphedema. Cureus.

